# Fission yeast cells use distinct cell size control mechanisms to regulate cell geometry in response to osmotic, oxidative, or low glucose conditions

**DOI:** 10.1091/mbc.E26-02-0064

**Published:** 2026-03-18

**Authors:** Elena J. Cabral, Pablo Andres, Geraldin Argandona, Paige Duggan, Benjamin M. Kuran, Kristi E. Miller

**Affiliations:** ^1^Biology Department, Providence College, Providence, RI 02918; NYU Grossman School of Medicine

## Abstract

Cells maintain an appropriate size to function, yet the mechanisms that enable size adaptation to environmental stress remain poorly understood. Fission yeast cells enter mitosis and divide at a threshold size when cyclin-dependent kinase (Cdk1) is activated through size- and time-dependent scaling of its regulators: Cdr2 kinase with cell surface area (SA), Cdc25 phosphatase with cell volume, and mitotic cyclin Cdc13 with time. This integrated size control network is characterized in nutrient-rich conditions, but under stress, it remains unclear which size parameters cells monitor, and which size- or time-sensing pathways mediate cell size changes. Using high-throughput image analysis, we quantified the geometry of dividing cells under osmotic, oxidative, and low glucose conditions. Wild-type cells increased their SA-to-volume (SA:Vol) ratio in low glucose but decreased it under osmotic or oxidative stress, revealing distinct stress-specific geometric responses. Genetic perturbations of size- and time-sensing pathways revealed that Cdc25 is required for volume-based expansion in oxidative and osmotic stress, Cdr2 promotes SA-based expansion in low glucose, and Cdc13 contributes to geometry changes under low glucose and osmotic stress. Although disrupting individual pathways altered normal geometric responses, cells remained viable, suggesting that a modular size control system enables flexible geometric responses to changing environments.

## Abstract (executive summary)

- Cells must regulate their size to function properly, yet how environmental stress reshapes cell geometry and the mechanisms that control cell size remain poorly understood.
- Using quantitative image analysis in fission yeast, we show that different stresses produce distinct changes in cell geometry that are mediated by specific size control pathways involving Cdr2, Cdc25, or Cdc13, which sense surface area, volume, and time, respectively.
- These findings reveal that cell size control is modular and flexible, providing a framework for understanding how conserved cell cycle regulators coordinate geometry under changing environmental conditions.

## INTRODUCTION

Cell size is a fundamental property that influences metabolism and gene expression and must be precisely regulated to ensure proper cell function. Most eukaryotic cells control their size by delaying cell cycle transitions until they reach a threshold size, indicating that growth and cell cycle progression are tightly coordinated (Turner *et al.,* 2012; Amodeo and Skotheim, 2016). However, cell size is not fixed. Environmental factors influence the size of cells in both unicellular and multicellular organisms. For example, starvation leads to reduced cell size in *Drosophila*, rats, and yeast (Robertson, 1963; Hirsch and Han, 1969; Fantes and Nurse, 1977; Johnston *et al.,* 1979). The ability to adjust cell size in response to environmental conditions is critical to maintain cellular function and survival, yet the molecular mechanisms that enable cells to adjust their size remain poorly understood (Kellogg and Levin, 2022).

**FIGURE 1:**
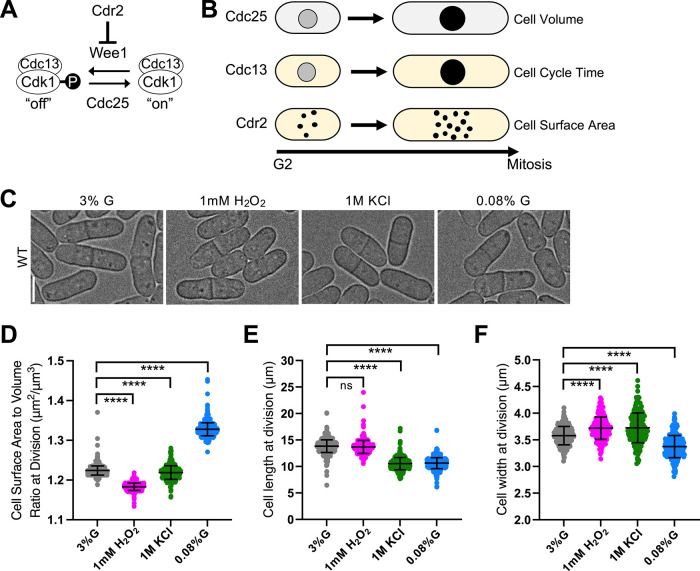
WT cells adjust their SA:Vol ratio based on stress condition. (A) Schematic of biochemical pathway regulating mitotic entry and cell size. (B) Cdk1 activators scale with distinct aspects of size or time. (C) Brightfield images of WT cells grown under various environmental conditions. (D) SA:Vol ratio at division. *n* > 300 for each condition. (E) Cell length at division. *n* > 300 for each condition. (F) Cell width at division. *n* > 200 for each condition. ns, not significant; ****p < 0.0001.

Fission yeast *Schizosaccharomyces pombe* is a powerful model system to investigate size control mechanisms due to its genetic tractability and simple rod shape. These cells grow by linear extension and divide at a reproducible size that has historically been measured by cell length but is now more accurately defined by surface area (SA) (Mitchison and Nurse, 1985; Pan *et al.,* 2014; Facchetti *et al.,* 2019). The core regulators of mitotic entry and cell size in fission yeast are conserved across eukaryotes (Figure 1A). Entry into mitosis is triggered by activation of the cyclin-dependent kinase Cdk1 (also called Cdc2 in fission yeast) bound to its mitotic cyclin, Cdc13. The kinase Wee1 inhibits Cdk1 in small cells, and the phosphatase Cdc25 activates Cdk1 once cells reach a threshold size (Russell and Nurse, 1987; Gould and Nurse, 1989). Recent work has shown that Cdk1 activation integrates size- and time-dependent inputs: the Wee1-inhibitory kinase Cdr2 cortical node density scales with cell SA (Pan *et al.,* 2014; Facchetti *et al.,* 2019); Cdc25 nuclear concentration scales with cell volume; and cyclin Cdc13 accumulates over time in the nucleus (Miller *et al.,* 2023) (Figure 1B). These size- and time-dependent properties of Cdk1 activators define a modular size control network that coordinates mitotic entry with cell growth through multiple pathways.

Although these cell SA- (Cdr2), volume- (Cdc25), and time-sensing (Cdc13) pathways have been characterized under nutrient-rich conditions in fission yeast (Pan *et al.,* 2014; Facchetti *et al.,* 2019; Miller *et al.,* 2023), their roles under environmental stress remain unclear. Stress conditions such as glucose limitation and osmotic or oxidative stress profoundly alter growth, morphology, and cell size (Kellogg and Levin, 2022; Chadha *et al.,* 2024), raising the question of which geometric parameters cells monitor to divide and which size- or time-sensing pathways drive stress-dependent size changes.

Here, we examine how fission yeast cells change their geometry under low glucose, oxidative stress, and osmotic stress. We show that wild-type (WT) cells adopt distinct geometric strategies, favoring SA expansion under glucose limitation and volume expansion under oxidative and osmotic stress. Genetic perturbations of *cdc25*, *cdc13*, and *cdr2* reveal that each pathway makes unique, stress-specific contributions to geometric changes. These findings support a model in which a modular size control network enables flexible geometric responses under changing environmental conditions.

## RESULTS AND DISCUSSION

To investigate how distinct cell size control pathways contribute to stress-dependent size changes, we analyzed WT and fission yeast mutants in which the SA-, volume-, or time-sensing pathways were disrupted (described below). Cells were grown for 48 h in YE4S media (3% glucose) as a control or under stress-inducing conditions: low glucose (0.08% glucose), oxidative stress (1 mM H_2_O_2_), or osmotic stress (1 M KCl) to capture long-term responses. Cell size was quantified using an established image analysis pipeline (Miller *et al.,* 2023). From these data, we obtained cell width, length, SA, volume, and SA-to-volume (SA:Vol) ratio of dividing cells. For each genotype, cell geometric parameters were compared across stress conditions relative to the YE4S controls using one-way ANOVA.

### WT cells exhibit stress-specific changes in SA:Vol ratio

First, we examined how WT cells alter their geometry in response to environmental stress (Figure 1C). Under oxidative and osmotic stress, the SA:Vol ratio decreased, indicating greater cell volume expansion relative to SA (Figure 1D). However, we note that the reduction in SA:Vol under osmotic stress, while statistically significant, was modest in magnitude (−0.4%). These changes occurred through stress-specific alterations in cell dimensions. During oxidative stress, cells became wider but maintained their length (Figure 1, E and F). Under osmotic stress, overall cell size decreased through reduced length, accompanied by an increase in width (Figure 1, E and F). In contrast, under low glucose conditions, cells increased their SA:Vol ratio, indicating a shift toward maximizing SA relative to volume (Figure 1D). WT cells reduced both their length and width in low glucose (Figure 1, E and F). Although previous studies primarily examined cell length, our stress-specific geometric changes are consistent with prior observations, including no major cell size changes under oxidative stress (Degols *et al.,* 1996; Salat-Canela *et al.,* 2021), reduced cell length under osmotic stress (Shiozaki and Russell, 1995), and coordinated changes in length, width, and SA:Vol ratio under low glucose conditions (Fantes and Nurse, 1977; Bertaux *et al.,* 2023).

These results show that WT cells adopt environment-specific geometric changes, preferably expanding in volume under oxidative and osmotic stress and in SA under glucose limitation. Even modest geometric changes can be biologically meaningful in fission yeast, where small cell size means subtle shifts in SA:Vol ratio translate into measurable changes in membrane area relative to cytoplasmic volume. Such adjustments may reflect physiological tuning that supports adaptation to distinct environments (Amodeo and Skotheim, 2016).

### Cdc25 is required for cell volume expansion under oxidative and osmotic stress

To test the role of Cdc25 in stress-dependent geometry changes, we examined mutants that disrupt either Cdc25 protein abundance or its size-dependent expression. Because Cdc25 nuclear concentration normally scales with cell volume (Miller *et al.,* 2023), we hypothesized that disrupting this scaling would impair volume expansion under oxidative and osmotic stress.

**FIGURE 2:**
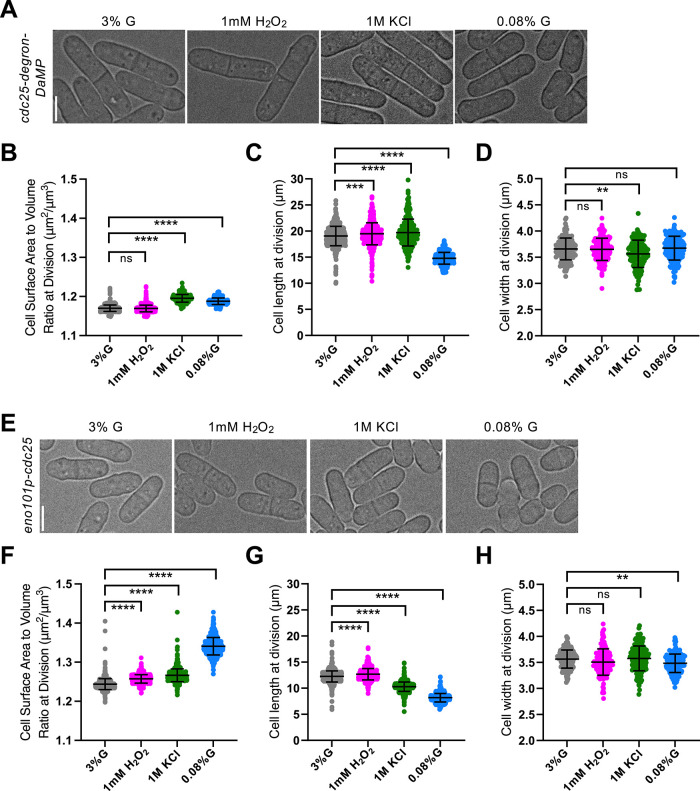
Cdc25 is necessary for maximizing cell volume under oxidative and osmotic stress. (A) Brightfield images of *cdc25-degron-DaMP* cells grown under various environmental conditions. (B) SA:Vol ratio at division. *n* > 250 for each condition. (C) Cell length at division. *n* > 250 for each condition. (D) Cell width at division. *n* > 150 for each condition. (E) Brightfield images of *eno101p-cdc25* cells grown under various environmental conditions. (F) SA:Vol ratio at division. *n* > 650 for each condition. (G) Cell length at division. *n* > 650 for each condition. (H) Cell width at division. *n* > 150 for each condition. ns, not significant; ****p < 0.0001; ***p < 0.001; **p < 0.01.

To reduce Cdc25 protein levels, we used a degron-DaMP–tagged allele of *cdc25* (Breslow *et al.,* 2008). Under oxidative and osmotic stress, *cdc25-degron-DaMP* cells failed to maximize volume like WT cells (Figure 2, A and B). Instead, in oxidative stress, these mutant cells maintained an SA:Vol ratio similar to the 3% glucose control and increased in size primarily by elongating, while cell width remained unchanged (Figure 2, B−D). Under osmotic stress, *cdc25-degron-DaMP* cells exhibited an ∼2% increase in SA:Vol ratio, opposite to the modest decrease observed in WT cells. This increase arose from increased cell length and reduced width (Figure 2, B−D). In contrast, under low glucose conditions, *cdc25-degron-DaMP* cells adapted similar to WT, reducing length and increasing SA:Vol ratio (Figure 2, B−D).

Because *cdc25-degron-DaMP* cells divide at a larger size than WT (Supplemental Figure S1A), we asked whether their altered geometry under oxidative and osmotic stress arose from disrupted Cdc25 volume-scaling or larger division size. To distinguish between these possibilities, we created an *eno101p-cdc25* strain, in which *cdc25* expression is driven by a constitutive *eno101* promoter (Wang *et al.,* 2014) rather than its native, size-dependent promoter (Keifenheim *et al.,* 2017). *eno101p-cdc25* cells exhibit strongly diminished size-dependent nuclear accumulation of Cdc25 (Supplemental Figure S1, B−D) and divide at a reduced size than WT (Supplemental Figure S1A). Under oxidative and osmotic stress, *eno101p-cdc25* cells also failed to maximize volume and instead relied on SA expansion (Figure 2, E and F), similar to *cdc25-degron-DaMP* cells. During oxidative stress, cells elongated without changing width, while osmotic stress caused them to become shorter while maintaining width (Figure 2, G and H). Under low glucose conditions, *eno101p-cdc25* cells responded like WT, increasing their SA:Vol ratio and decreasing both length and width (Figure 2, E–H).

Together, these results show that disrupting either Cdc25 protein abundance or size-dependent expression shifts cells from a volume- to an SA-based geometry strategy. Proper Cdc25 scaling with volume thus ensures coupling between cell volume and mitotic entry, allowing cells to adjust their size in response to oxidative or osmotic stress.

### Time-sensing pathway (Cdc13) supports proper size regulation under low glucose and osmotic stress

To access the role of the Cdc13 time-sensing pathway, we disrupted Cdc13 protein abundance by creating a *cdc13-2x* strain carrying an additional copy of *cdc13* integrated at an exogenous locus. *cdc13-2x* cells were divided at a smaller size than WT, reflecting accelerated mitotic entry upon disruption of the typical time-scaling of Cdc13 protein (Supplemental Figure S1A).

**FIGURE 3:**
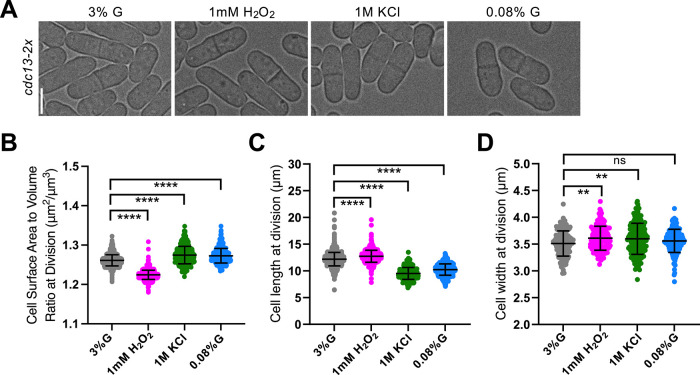
Cdc13 time-sensing pathway contributes to cell geometry changes under low glucose and osmotic stress. (A) Brightfield images of *cdc13-2x* cells grown under various environmental conditions. (B) SA:Vol ratio at division. *n* > 425 for each condition. (C) Cell length at division. *n* > 425 for each condition. (D) Cell width at division. *n* > 150 for each condition. ns, not significant; ****p < 0.0001; **p < 0.01.

Under oxidative stress, *cdc13-2x* cells responded similarly to WT. These cells increased in both length and width, resulting in a decrease in the SA:Vol ratio (Figure 3, A−D). Under low glucose, *cdc13-2x* cells also showed a WT-like response, decreasing length and increasing the SA:Vol ratio (Figure 3, B−D). However, this SA:Vol increase was reduced in magnitude compared with WT (1 vs. 8.5%; Figures 1D and 3B), suggesting impaired geometric remodeling under glucose limitation.

In contrast, under osmotic stress, *cdc13-2x* cells failed to maximize cell volume (Figure 3B). These cells moderately decreased in length and increased in width, leading to an overall increase in the SA:Vol ratio (Figure 3, B−D), opposite to WT cells. Notably, osmotic and low glucose conditions were the only stress conditions that showed significantly reduced doubling times relative to control 3% glucose media (Supplemental Figure S3E), potentially increasing reliance on Cdc13’s time-sensing function to coordinate mitotic timing with growth. Together, these results suggest that Cdc13 contributes to stress-dependent control of cell geometry under low glucose and osmotic stress conditions.

### The Cdr2 cell SA-sensing pathway supports SA maximization under low glucose conditions

**FIGURE 4:**
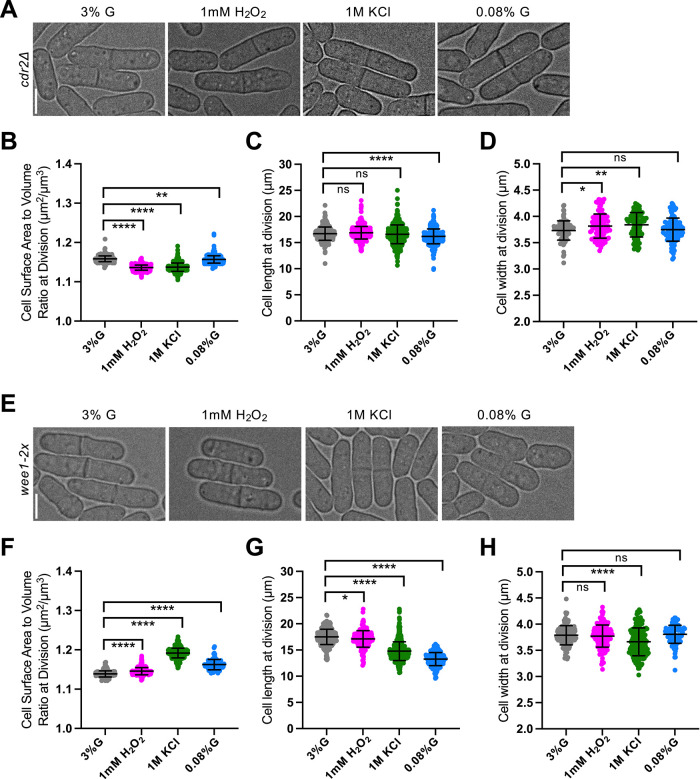
*cdr2Δ* cells fail to maximize SA in low glucose, while *wee1-2x* mutants increase SA:Vol ratio under all stress conditions. (A) Brightfield images of *cdr2Δ* cells grown under various environmental conditions. (B) SA:Vol ratio at division. *n* > 400 for each condition. (C) Cell length at division. *n* > 400 for each condition. (D) Cell width at division. *n* > 100 for each condition. (E) Brightfield images of *wee1-2x* cells grown under various environmental conditions. (F) SA:Vol ratio at division. *n* > 400 for each condition. (G) Cell length at division. *n* > 400 for each condition. (H) Cell width at division. *n* > 100 for each condition. ns, not significant; ****p < 0.0001; **p < 0.01; *p < 0.04.

Cdr2 functions as a cortical scaffold that regulates Wee1 activity in relation to cell size, with Cdr2 nodal density reported to scale with cell SA, enforcing an SA threshold for mitotic entry (Pan *et al.,* 2014; Allard *et al.,* 2018; Facchetti *et al.,* 2019; Sayyad and Pollard, 2022). We hypothesized that Cdr2 might be necessary for the SA-based expansion observed under low glucose. To test this, we analyzed *cdr2Δ* cells (Figure 4A).

Under oxidative and osmotic stress, *cdr2Δ* cells responded similarly to WT, decreasing their SA:Vol ratio through modest width increases with little change in cell length (Figure 4, B−D), indicating that Cdr2 is dispensable for volume-based changes. In contrast, under low glucose conditions, *cdr2Δ* cells failed to maximize SA and instead showed a slight reduction in the SA:Vol ratio, opposite to WT (Figure 4B). This defect arose from a smaller decrease in cell length (0.52 µm for *cdr2Δ* vs. 3.2µm for WT) and no change in width (Figure 4, C and D). These results suggest that Cdr2 promotes SA-based expansion during glucose limitation, likely by enforcing an SA threshold for division in low glucose environments.

To determine whether these effects reflect a specific role of Cdr2 or altered Wee1 regulation, we examined a *wee1-2x* mutant carrying an additional copy of *wee1* at an exogenous locus (Figure 4E). Interestingly, *wee1-2x* cells increased their SA:Vol ratio under all stress conditions (Figure 4F). These cells decreased in length under oxidative, osmotic, and low glucose conditions, with no change in width except a decrease under osmotic stress (Figure 4, G and H). Elevated *wee1* levels lead to more inactive Cdk1, causing *wee1-2x* cells to divide at a larger size (Miller *et al.,* 2023) (Supplemental Figure S1A). Mitotic entry in these cells likely requires additional Cdr2-mediated suppression, and the resulting delay in division may allow for greater SA accumulation. Together, these findings identify Cdr2 as a key regulator of SA-based geometric changes under glucose limitation.

### Defects in SA or volume expansion do not uniformly compromise growth or survival

To determine whether disruption of normal stress-induced cell geometry impacts cell growth or viability under stress, we examined septation index values and population growth rates. Septation frequencies were largely similar to WT, except under low glucose, where *cdr2Δ* and *wee1-2x* cells showed altered septation consistent with perturbed SA-based regulation (Supplemental Figure S2).

Doubling times were highly consistent across genotypes in 3% glucose YE4S (136 ± 7 min; ∼12% variation) and under low glucose conditions (163 ± 8 min; ∼13% variation), despite slowing of growth (Supplemental Figure S3). Oxidative stress modestly increased genotype-dependent variation (135 ± 9 min; ∼18% variation), but osmotic stress showed the strongest genotype dependence (241 ± 19 min; ∼23% variation). Strains with prolonged doubling times under osmotic stress, including *cdc13-2x*, *cdc25-degron-DAmP*, and *wee1-2x*, also failed to maximize cell volume like WT cells. However, *eno101p-cdc25* showed similar geometric defects in osmotic stress yet maintained doubling times comparable with WT (Supplemental Figure S3E).

Together, these results indicate that impaired SA or volume expansion does not uniformly compromise growth or survival. Future studies tracking cell populations through chronic or recurring stress will be needed to clarify how these geometric changes shape long-term proliferation, recovery, and evolutionary resilience. Changes in growth rate or cell cycle progression under stress may influence division size by altering mitotic regulator accumulation. Future studies measuring mitotic regulator dynamics alongside growth rates will be needed to distinguish direct changes in size control mechanisms from growth- or timing-dependent effects.

The stress-specific size phenotypes observed here highlight how size control pathways intersect with stress signaling. To our knowledge, stress-induced cell size changes have not been systematically characterized for the *cdc25*, *cdr*2, and *cdc13* mutants examined. *cdc25* mutants elongated under osmotic and oxidative stress, consistent with Cdc25’s previously reported role in the Sty1 MAP kinase pathway, which mediates response to these stresses (Millar *et al.,* 1995; Shiozaki and Russell, 1995; Degols *et al.,* 1996; Shiozaki *et al.,* 1998; López-Avilés *et al.,* 2005; 2008). Our data suggest that Cdr2 likely supports SA expansion in low glucose. Cdr2 forms cortical nodes whose activity and localization are modulated by the Pom1 gradient, which itself is responsive to glucose availability (Kelkar and Martin, 2015; Allard et al., 2018). Consistent with our observation that Cdc13 contributes to osmotic stress-dependent size regulation, *cdc13* expression is regulated by the Sty1 downstream transcription factor Atf1 (Wilkinson *et al.,* 1996; Bandyopadhyay *et al.,* 2014). Future studies will be needed to determine how conserved stress-responsive pathways (Sty1-Srk1, PKA, and TOR) regulate Cdc25-, Cdc13-, and Cdr2-scaling properties and to distinguish transient from long-term stress response mechanisms.

**FIGURE 5:**
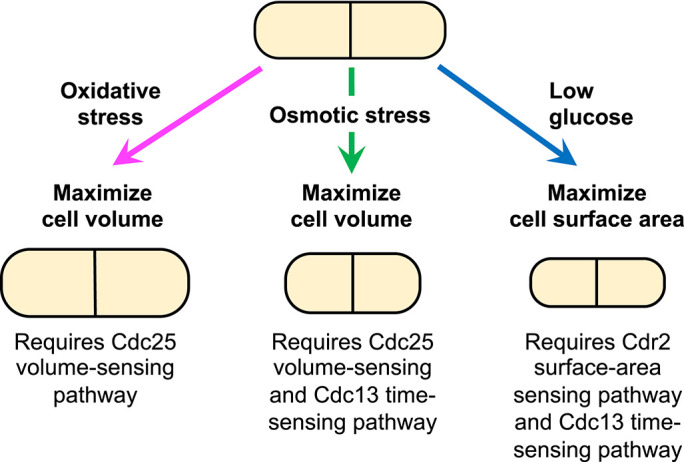
Distinct size control pathways mediate stress-specific geometric changes in fission yeast. Cdc25, Cdc13, and Cdr2 mediate distinct geometric responses to oxidative, osmotic, and low glucose stress, enabling fission yeast to flexibly adapt cell size for robust division under changing environments.

Collectively, our results show that fission yeast alter their cell geometry by maximizing either SA or volume depending on environmental conditions. Volume-based changes in oxidative stress require Cdc25, osmotic stress size regulation requires both Cdc25 and Cdc13, and SA-based changes in low glucose depend on Cdr2 and Cdc13 (Figure 5). When one pathway is disrupted, cells often switch to an alternate geometric mode, revealing flexibility within the size control network. Together, these findings suggest that an integrated size control system allows cells to dynamically reweight size- and time-sensing mechanisms to maintain robust Cdk1 regulation and size homeostasis in changing environments.

## MATERIALS AND METHODS

Request a protocol through *Bio-protocol*

### Strain construction and growth conditions

Standard methods were used to grow *S. pombe* cells (Moreno *et al.,* 1991). Yeast strains used in this study are listed in Supplemental Table S1. Gene fusions were expressed under their endogenous promoters unless noted. PCR-based homologous recombination was performed for tagging of genes on the chromosome (Bähler *et al.,* 1998). To place *cdc25* under the control of the constitutive *eno101* promoter, we synthesized a gBlock fragment (Integrated DNA Technologies) containing the *kanMX6* marker and a 276 bp *eno101p* sequence (Wang *et al.,* 2014). The *kanMX6-eno101p* cassette was PCR-amplified and integrated at the 5′ end of the *cdc25* open reading frame on the chromosome using standard N-terminal tagging methods (Bähler *et al.,* 1998). This strain was confirmed to have significantly reduced size-dependent accumulation of Cdc25 by analysis of fluorescent protein intensity (Supplemental Figure S1, B−D), described below. To obtain *cdc13-2x* cells carrying an additional copy of *cdc13+,* we integrated the *StuI*-linearized *pHis5StuI-cdc13-sfGFPint* plasmid (a kind gift of Jamie Moseley) at the *his5* locus of *his5-D21* yeast cells. This strain was confirmed to be correct by growth on EMM—his plates and detection of Cdc13-sfGFPint signal by fluorescent microscopy.

Cells were cultured in standard YE4S medium (3% glucose) overnight and then maintained or shifted to stress conditions. Oxidative stress was induced by supplementing YE4S with 1 mM hydrogen peroxide, osmotic stress with 1 M KCl, and glucose limitation with 0.08% glucose. These stress intensities were selected based on prior studies demonstrating robust cellular responses while preserving cell viability and the capacity for recovery (Fantes and Nurse, 1977; Shiozaki and Russell, 1995; Degols *et al.*, 1996; López-Avilés *et al.*, 2008; Sansó *et al.*, 2008; Saitoh et al., 2015; Salat-Canela *et al.*, 2021; Bertaux *et al.*, 2023).

Cultures were incubated at 25°C while shaking at 180 rpm for 48 h before imaging. This timepoint was chosen to ensure that cells had progressed beyond the initial transient stress response and reached a stable, condition-specific growth state, as confirmed by growth curve and doubling time analyses showing stabilization well before 48 h, including in the slowest growing condition (1 M KCl; doubling time ∼4 h) (Supplemental Figure S3). Each yeast strain was grown in triplicate under each condition to ensure reproducibility of cell size measurements. After confirming consistency across individual trials, datasets were combined and plotted together.

### Microscopy

#### Analysis of cell geometry from static images

Cells were harvested by brief centrifugation, resuspended, and ∼2 µl of concentrated suspension was spotted onto 35-mm glass-bottom dishes (P35G-1.5-20C; MatTek) overlaid with YE4S agar (prepared with the same condition as the culture medium and prewarmed to 25°C). Imaging was performed using a widefield epifluorescence microscope: Nikon Ti2-A inverted microscope (NIS-Elements software) equipped with a Prior Z-motorized focus drive; a 60 × 1.4 NA CFI60 Plan Apochromat Lambda D oil-immersion objective lens (Nikon); a D-LEDi fluorescence LED illumination system with DAPI, GFP, and mCherry filter cubes; and a PCO.panda USB 3.1 sCMOS camera. For each cell type and condition, multiple fields of view were imaged. Brightfield and DAPI images were captured with 27 optical z-sections and 0.2-µm step size. Cell images displayed in figures are brightfield single z-section images, and scale bars are 5 µm.

#### Analysis of fluorescent protein intensity from static images

For imaging to examine size-dependent accumulation of *eno101p-cdc25-mNeonGreen* compared with WT *cdc25-mNeonGreen*, cells were mounted on a coverglass-bottom dish (P35G-1.5-20C; MatTek) and covered with a piece of YE4S (3% glucose) agar prewarmed to 25°C. Imaging was performed using a spinning disk confocal microscope (Yokogawa CSU-WI; Nikon Software) equipped with a 60 × 1.4-NA CFI60 Plan Apochromat Lambda D oil-immersion objective lens (Nikon); 405-, 488-, and 561-nm laser lines; and a Photometrics Prime BSI camera on an Eclipse Ti2 inverted microscope (Nikon). Multiple fields of view were acquired for each cell type, with 27 optical z-sections collected at 0.2-µm intervals.

### Cell and nuclei segmentation

Cell and nuclei segmentation was carried out using a semiautomated pipeline as described in Miller *et al.,* 2023. In brief, nuclear segmentation was based on the BFP-NLS signal, which reliably marked nuclei, while Brightfield images were used for cell segmentation. In ImageJ, Brightfield stacks were smoothed with Gaussian filtering, followed by global thresholding to generate binary masks of cell outlines. Morphological operations (erosion and dilation) were used to refine masks and separate adjacent cells. Artifacts, edge cells, and unresolved clumps of cells were removed manually in ImageJ. The resulting binary image (“cell mask”) was compared with the original brightfield image and confirmed to be an accurate representation of cell size.

BFP-NLS images were processed for nuclear segmentation using a semiautomated ImageJ pipeline (as in Miller *et al.,* 2023). In ImageJ, a sum projection was created from z-stacks and was subsequently smoothed and globally thresholded to generate binary nuclear masks. Masks were filtered to exclude edge objects. To correct for uneven BFP illumination across an imaging field, abnormally small or large nuclei were removed from binary masks. The resulting binary masks (“nuclei mask”) were then validated against the original BFP-NLS images to ensure accurate nuclear size representation.

Cell masks of dividing cells were generated using ImageJ and MATLAB as described in Miller *et al.,* 2023. Briefly, nuclear masks were eroded in ImageJ to separate nuclei from cell borders, then combined with cell masks in MATLAB to produce nuclei-overlaid images. These were filtered to retain only cells containing two nuclei, manually confirmed, and further processed to remove nuclei, yielding binary masks of dividing cells.

### Cell geometry measurements

Cell geometry measurements were obtained as described in Miller *et al.,* 2023. In brief, cell widths (≥50 cells per condition) were manually measured from cell masks in ImageJ using the straight-line tool, avoiding irregular regions. This approach provided more reproducible measurements for SA and volume calculations than automated methods (i.e., using ImageJ or MATLAB). For each strain and condition, the mean cell radius (defined as average cell width/2) was used to calculate cell SA and volume, assuming a uniform radius across the population (Table 1). Cell length or symmetry axes (defined as the total tip-to-tip distance along the long axis of each cell) were identified in MATLAB by applying principal component analysis of the cloud points within each segmented cell (Facchetti *et al.,* 2019). Cell SA and volume were calculated in MATLAB using the equation for a cylinder with hemispherical ends, reflecting the rod-like geometry of fission yeast (Table 1) (Navarro and Nurse, 2012; Pan *et al.,* 2014; Facchetti *et al.,* 2019; Baybay et al., 2020; Miller *et al.,* 2023). Cell volume and SA values obtained here were consistent with previously published work (Facchetti *et al.,* 2019; Miller *et al.,* 2023).

**TABLE 1:** Calculation of cell SA or volume.

Name	“Mean radius of cell population”	“Rotation”
Description	Using the average radius of a cell population determined by manual measurements of individual cells	Rotation around the symmetry axis of each cell
Cell radius	*R*	Function *R*(x)^a^
Cell length	*L*	*L*
Cell surface area	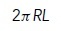	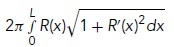
cell volume	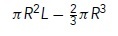	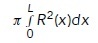

To validate our cell size measurements and confirm the robustness of our results, we also calculated the SA and volume of cells by rotation of the *R*(x) function (defined as the shortest distance from the cell border to the symmetry axis, *R*(x), 0 ≤ x ≤ *L*) of each single cell around the symmetry axis. This method avoids assuming a cylindrical cell shape (Table 1; as done by Facchetti *et al.,* 2019, and utilized in Miller *et al.,* 2023). We compared the cell size results obtained from this rotation method and the mean radius of cell population method for all datasets. Both approaches gave consistent results and supported the same conclusions. SA:Vol ratios calculated using the rotation method are shown in Supplemental Figure S1.

### Measurement of fluorescent intensity

Summed projections of z-sections were used to analyze fluorescent intensity. MATLAB scripts quantified mean fluorescence of proteins in the cytoplasm, nucleus, or whole cell, as defined by cell and nuclear masks.

All MATLAB codes used for image processing and analysis are available on GitHub (https://github.com/millerk89/sizer-timer-pombe), as previously archived in Miller *et al.,* 2023.

### Septation index analysis

Brightfield images of cells, taken from the same datasets used for cell size measurements, were analyzed to determine septation index. For each strain and condition, at least 100 cells were counted to calculate the percentage of cells with a single septum versus no septum. Statistical comparisons were performed using  *χ*^2^ tests on raw cell counts in GraphPad Prism. For each condition, an overall *χ*^2^ test was first used to assess differences in septation index across genotypes. When a significant effect was detected, pairwise *χ*^2^ tests were performed comparing individual mutant strains with WT.

### Growth curve and doubling time analysis

Cells were grown to mid-log phase in YE4S 3% glucose media and then switched to proper control or stress media (YE4S 3% glucose media or with 1 mM hydrogen peroxide, 1 M KCl, or with 0.08% glucose) and diluted to an OD value of 0.05. A total of four replicates were plated in a 96-well plate (i.e., media switching was performed right before cells were plated). Cells were grown at 32°C while shaking for 33 h in a BioTek Cytation 3 plate reader, and the OD_600nm_ of each well was taken every 2 min. Doubling time was calculated from exponential growth rate constants (*k*) obtained by nonlinear regression of each replicate growth curve using a sigmoidal model (GraphPad Prism), where doubling time = *ln*(2)/*k*.

### Statistical analysis

Data analysis was performed in GraphPad Prism. Data are presented as mean values with SD unless otherwise noted. Comparisons among multiple strains or growth conditions were assessed by ANOVA. Comparisons between two groups were assessed using an unpaired two-tailed *t* test. The *p*-values of <0.05 were considered statistically significant. Linear regression analysis was used to obtain slope values for assessing the relationship between cell volume and Cdc25 mean intensity in the nucleus for WT and *eno101p-cdc25* cells.

## Supporting information





## Abbreviations used:

BFP: blue fluorescent protein
mNG: mNeonGreen
NLS: nuclear localization sequence
SA: surface area
SA:Vol: SA-to-Vol
Vol: volume
WT: wild-type.
