# Supplementary material for: Fission yeast cells use distinct cell size control mechanisms to regulate cell geometry in response to osmotic, oxidative, or low glucose conditions
Source: Mol Biol Cell. 2026 Mar 18;37(4):br11. doi: 10.1091/mbc.E26-02-0064 (PMC13008265; doi:10.1091/mbc.E26-02-0064)
Supplement: Supplementary file 1 [file mbc-37-br11-s001.pdf]

Supplemental Materials

Molecular Biology of the Cell

Cabral *et al*.

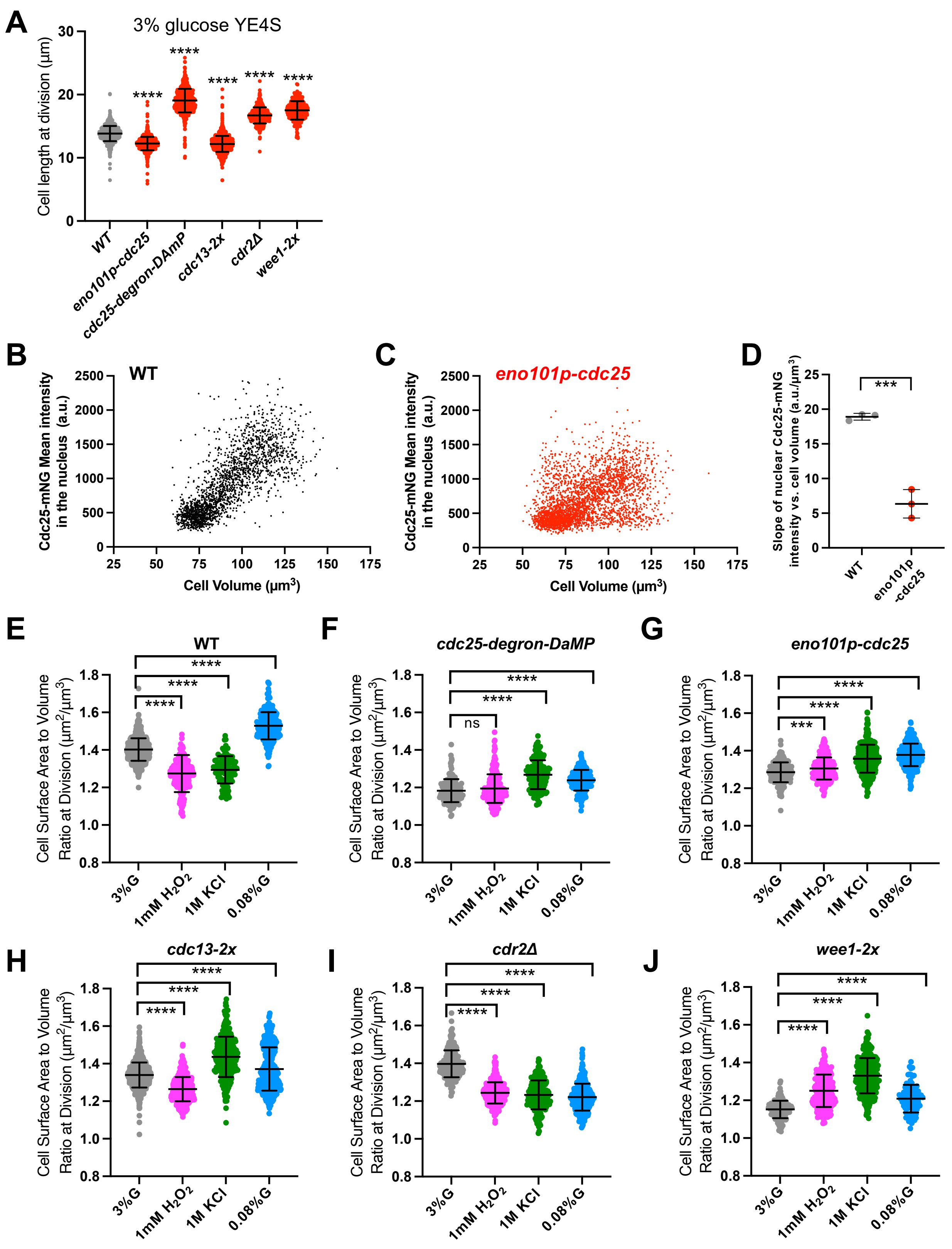

A
3% glucose YE4S
Cell length at division (μm)
0
10
20
30
****
****
****
****
****
WT
eno101p-cdc25
cdc25-degron-DAmP
cdc13-2x
cdr2Δ
wee1-2x
B
WT
Cdc25-mNG Mean intensity in the nucleus (a.u.)
Cell Volume (μm³)
C
eno101p-cdc25
Cdc25-mNG Mean intensity in the nucleus (a.u.)
Cell Volume (μm³)
0
500
1000
1500
2000
2500
25
50
75
100
125
150
175
D
Slope of nuclear Cdc25-mNG intensity vs. cell volume (a.u./μm³)
***
WT
eno101p -cdc25
0
5
10
15
20
25
E
WT
F
cdc25-degron-DaMP
G
eno101p-cdc25
H
cdc13-2x
I
cdr2Δ
J
wee1-2x
Cell Surface Area to Volume Ratio at Division (μm²/μm³)
0.8
1.0
1.2
1.4
1.6
1.8
****
***
ns
3%G
1mM H₂O₂
1M KCl
0.08%G

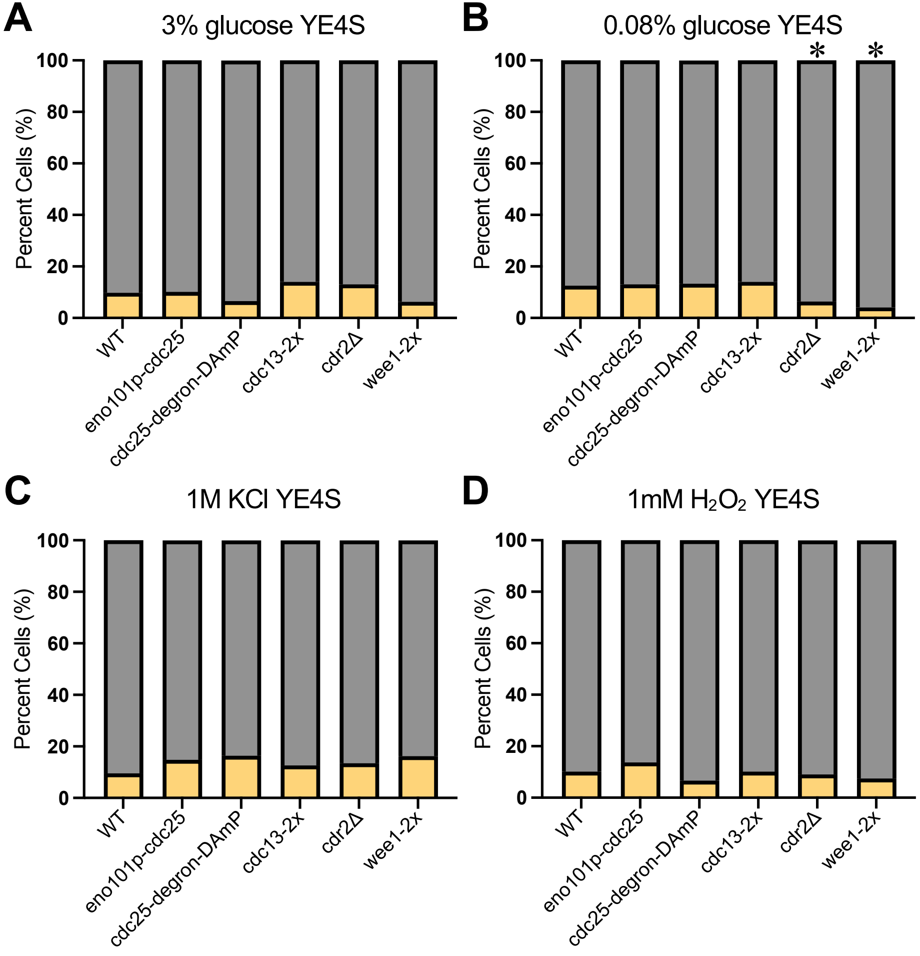
A
3% glucose YE4S
B
0.08% glucose YE4S
C
1M KCl YE4S
D
1mM H_2O_2 YE4S
Percent Cells (%)
0
20
40
60
80
100
*
*
WT
eno101p-cdc25
cdc25-degron-DAmP
cdc13-2x
cdr2Δ
wee1-2x

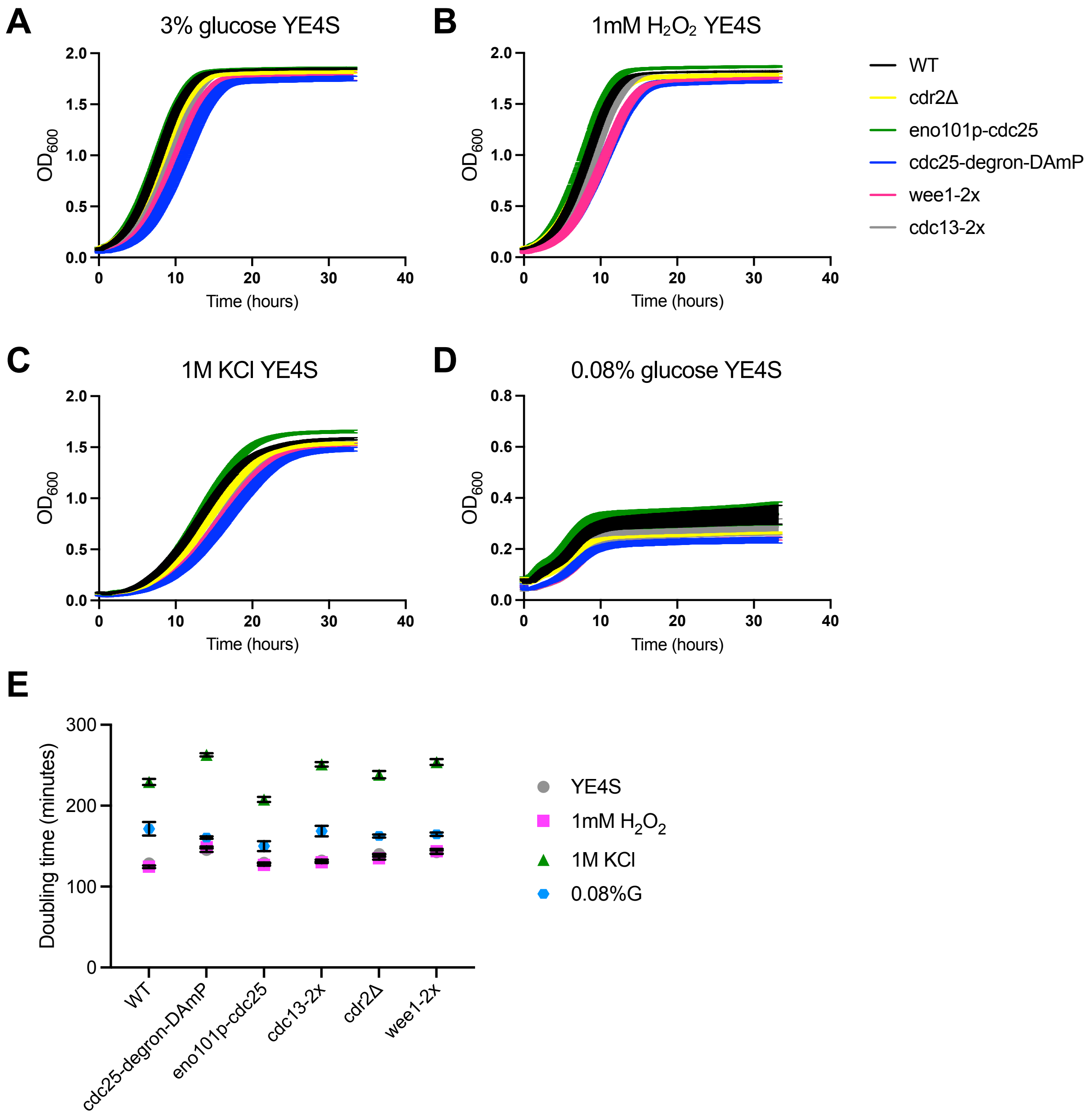
A
3% glucose YE4S
B
1mM H_2O_2 YE4S
C
1M KCl YE4S
D
0.08% glucose YE4S
E
OD_{600}
Time (hours)
WT
cdr2Δ
eno101p-cdc25
cdc25-degron-DAmP
wee1-2x
cdc13-2x
Doubling time (minutes)
YE4S
1mM H_2O_2
1M KCl
0.08%G

Figure S1: Verification of fission yeast genotypes and cell size measurements. (A) Cell length at division. All ****$p < 0.0001$ compared to WT. (B) Cdc25-mNG nuclear mean intensity in WT cells; n=2,133 or (C) *eno101p-cdc25* cells; n=3,160 grown in 3% glucose YE4S media. (D) Slopes from linear regression of three independent trials examining Cdc25 nuclear mean intensity vs. cell volume in WT or *eno101p-cdc25* cells. Each trial n> 500 cells. (E-J) Size measurements were verified by a rotation method (see methods) that confirmed the robustness of our results. Each condition: WT, n>100; *cdc25-degron-DaMP,* n>150; *eno101p-cdc25,* n>250; *cdc13-2x,* n>290; *cdr2Δ,* n>175; *wee1-2x,* n>90. ****$p < 0.0001$; *** $p<0.001$; ns, not significant.

Figure S2: Septation index of fission yeast strains grown under various conditions. (A) Yellow bar, single septum; Grey bar, no septum. n ≥ 100 cells per strain and condition. Asterisks indicate significant differences from WT. *$p <0.004$.

Figure S3: Growth curve and doubling times of fission yeast strains grown under various conditions. (A-D) OD_{600} over time for six yeast strains (colors at right), grown in (A) 3% glucose, (B) 1mM H_2O_2, (C) 1M KCl, and (D) 0.08% glucose. Lines show mean ± SD from four replicates. (E) Doubling times calculated from the exponential growth phase. Symbols indicate mean ± SD.

Table S1. Yeast strains used in this study

Strain	Genotype	Source
**KMY127**	*lys3+:ptdh1*:NLS-linker-mTagBFP2:terminatortdh1:nat R ULA+ h-*	Lab collection
**KMY357**	*cdr2Δ::NAT lys3+:ptdh1*:NLS-linker-mTagBFP2:terminatortdh1:kanMX ULA+*	Lab collection
**KMY378**	*(AKA wee1-2x) lys3+:ptdh1*:NLS-linker-mTagBFP2:terminatortdh1:kanMX leu1-32:[pJK148-Pwee1-wee1-Twee1] UA+*	(Miller *et al.,* 2023)
**KMY368**	*kanMX6::eno101p-cdc25-mNeonGreen::hphR lys3+:ptdh1*:NLS-linker-mTagBFP2:terminatortdh1:natR h- ULA+*	This study
**KMY376**	*cdc25-degron-DAmP::kanMX6 lys3+:ptdh1*:NLS-linker-mTagBFP2:terminatortdh1:natR ULA+ h*	Lab collection
**KMY385**	*(AKA cdc13-2x) pHis5StuI-cdc13-sfGFPint lys3+:ptdh1*:NLS-linker-mTagBFP2:terminatortdh1:natR ULA+*	This study
**KMY349**	*his5-D21 h-*	Vještica *et al.*, 2020
